# Platelet count and abdominal dynamic CT are useful in predicting and screening for gastroesophageal varices after Fontan surgery

**DOI:** 10.1371/journal.pone.0257441

**Published:** 2021-10-07

**Authors:** Yoshiharu Isoura, Akira Yamamoto, Yuki Cho, Eiji Ehara, Atsushi Jogo, Tsugutoshi Suzuki, Yuga Amano-Teranishi, Kiyohide Kioka, Takashi Hamazaki, Yosuke Murakami, Daisuke Tokuhara

**Affiliations:** 1 Department of Pediatrics, Osaka City University Graduate School of Medicine, Osaka, Japan; 2 Department of Diagnostic and Interventional Radiology, Osaka City University Graduate School of Medicine, Osaka, Japan; 3 Department of Pediatric Cardiology, Osaka City General Hospital, Osaka, Japan; 4 Department of Pediatric Electrophysiology, Osaka City General Hospital, Osaka, Japan; 5 Department of Hepatology, Osaka City General Hospital, Osaka, Japan; Policlinico S. Orsola-Malpighi, ITALY

## Abstract

**Objective:**

Patients who undergo Fontan surgery for complex cardiac anomalies are prone to developing liver and gastrointestinal complications. In particular, gastroesophageal varices (GEVs) can occur, but their prevalence is unknown. We aimed to elucidate the occurrence of GEVs and the predicting parameters of GEVs in these patients.

**Materials and methods:**

Twenty-seven patients (median age, 14.8 years; median time since surgery, 12.9 years) who had undergone the Fontan surgery and were examined by abdominal dynamic computed tomography (CT) for the routine follow-up were included in the study. Radiological findings including GEVs and extraintestinal complications were retrospectively evaluated by experienced radiologists in a blinded manner. Relationships between blood-biochemical and demographic parameters and the presence of GEVs were statistically analyzed.

**Results:**

Dynamic CT revealed gastric varices (n = 3, 11.1%), esophageal varices (n = 1, 3.7%), and gastrorenal shunts (n = 5, 18.5%). All patients with gastric varices had gastrorenal shunts. All gastric varices were endoscopically confirmed as being isolated and enlarged, with indications for preventive interventional therapy. A platelet count lower than 119 × 10^9^ /L was identified as a predictor of GEV (area under the receiver operating curve, 0.946; sensitivity, 100%; and specificity, 87%).

**Conclusions:**

GEVs are important complications that should not be ignored in patients who have undergone a Fontan procedure. Platelet counts lower than 119 × 10^9^ /L may help to prompt patient screening by using abdominal dynamic CT to identify GEVs and their draining collateral veins in these patients.

## Introduction

**Fig 1:**
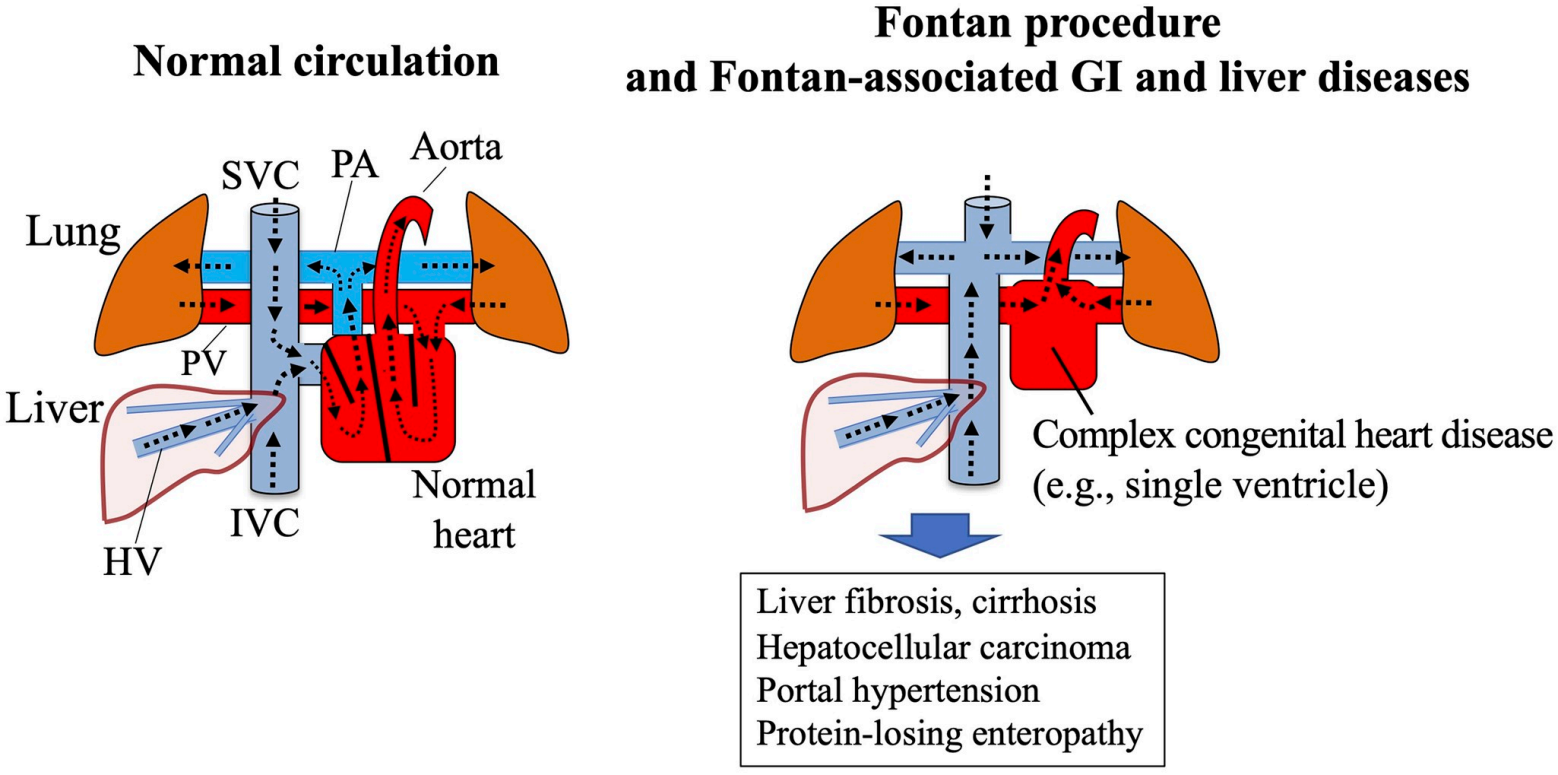
Fontan procedure and Fontan-associated liver diseases. After the Fontan procedure, the hepatic venous flow empties directly into the Fontan circuit; thus the liver is susceptible to the effects of central venous hypertension. High central venous pressure leading to chronic passive congestion occurs subsequent to the sinusoidal dilatation that follows increased intrahepatic resistance and reduced portal flow. Increased intrahepatic resistance promotes liver fibrogenesis, which manifests as Fontan-associated liver disease (e.g., cirrhosis and hepatocellular carcinoma). In addition, both increased intrahepatic resistance and reduced portal flow contribute to the development of portal hypertension. HV, hepatic vein; IVC, inferior vena cava; PA, pulmonary artery; PV, pulmonary vein; SVC, superior vena cava; GI, gastrointestinal.

The Fontan procedure connects the inferior vena cava to the pulmonary artery through a conduit or an intra-arterial baffle. With over 5000 operations annually worldwide it is one of the most frequently performed congenital heart surgeries for children with a functional single ventricle (Fig 1) [1–3]. Over the past several decades, perioperative and early mortality after the Fontan operation have decreased markedly because of beneficial surgical refinements [4]. However, over the long term, patients may develop liver fibrosis (mild to severe and cirrhosis; 0.85%–58.3% of patients [5–9], hepatocellular carcinoma (1.15%–3.4% [9–11]), or protein-losing enteropathy (2.5%–11% [12–15]).

The underlying pathophysiology of these complications may reflect the elevated central venous pressure and decreased cardiac output that follow the Fontan procedure. After surgery, the hepatic venous flow empties directly into the Fontan circuit, such that the liver is susceptible to the effects of central venous hypertension [16, 17]. High central venous pressure also leads to chronic passive congestion subsequent to sinusoidal dilatation, with increased intrahepatic resistance and reduced portal flow [16, 17]. Decreased cardiac output can further contribute to the reduction of portal flow. Increased intrahepatic resistance promotes liver fibrogenesis that manifests as Fontan-associated liver disease (e.g., cirrhosis and hepatocellular carcinoma). Both increased intrahepatic resistance and reduced portal flow promote the development of portal hypertension. These liver diseases and portal hypertension develop with increasing time since the patient underwent Fontan surgery [17]. A previous study demonstrated that >18 years since the Fontan procedure was associated with greater liver fibrosis than a time ≤18 years [18]. In a retrospective study using various imaging modalities, 90 of the 145 patients (62%) evaluated developed abnormalities, including liver heterogeneity, portal hypertension, and cirrhosis, at a mean of 27 years after the Fontan procedure [10]. Therefore, the evaluation and management of gastrointestinal and liver complications has emerged as an important issue not only for cardiologists and pediatricians but also for gastroenterologists, hepatologists and radiologists who follow these patients from childhood through adulthood.

Gastroesophageal varices (GEVs) can arise as a consequence of liver cirrhosis and portal hypertension [19] and have caused life-threatening events (e.g., rupture and bleeding) in some patients after the Fontan procedure [7, 20, 21]. However, the importance of screening for GEVs and the predictors of their occurrence in patients who have undergone Fontan procedure are not fully understood. To address these issues, we first aimed to clarify the occurrence of GEVs in Fontan patients by using abdominal dynamic CT, which is a useful non-endoscopic procedure for detecting GEVs [22, 23]. We then assessed whether various biochemical or demographic parameters predicted GEV occurrence in these patients.

## Materials and methods

### Study population

Patients who had received a Fontan procedure at least 5 years previously and were examined via contrast-enhanced abdominal CT by the dynamic bolus technique for the routine evaluation of abdominal complications from March 2013 through October 2019 were included in the study. The abdominal CT images were retrospectively evaluated for abdominal complications (e.g., liver tumors, collateral veins, and GEVs) by two experienced radiologists, who each had more than 15 years of experience in the interpretation of abdominal CT images. In addition to CT findings, we collected and analyzed demographic data (age, sex, and time since completion of the Fontan procedure) at the time of the CT examination, and laboratory tests (e.g., platelet counts, albumin, plasma ammonia, prothrombin time, Model for End-stage Liver Disease XI (MELD-XI) score, asparagine aminotransferase (AST), and alanine aminotransferase [ALT]) were obtained within 3 months of the CT examination. To examine the relationship between blood-biochemical parameters and the occurrence of GEVs, we calculated the AST-to-platelet ratio index (APRI) and fibrosis-4 index (FIB-4) values by using the following formulae, in accordance with previous studies [24–27]:

$$\text{APRI}=\left[\text{AST}\;\left(\text{IU}/\text{L}\right)/\text{upper}\;\text{limit}\;\text{of}\;\text{normal}\times 100\right]/\text{platelet}\;\text{count}\left(10^{9}/\text{L}\right),$$

and

$$\text{FIB-4}=\left[\text{age}\;\left(\text{years}\right)\times \text{AST}\left(\text{IU}/\text{L}\right)\right]/\left[\text{platelet}\;\text{count}\;\left(10^{9}/\text{L}\right)\times \text{ALT}\;{\left(\text{IU}/\text{L}\right)}^{1/2}\right]$$

[28, 29].

Patients who had serologic evidence of viral hepatitis B or C infection were excluded from the study.

Informed consent was obtained by the opt-out methodology. Briefly, a letter explaining the study using participants’ anonymous data and an opt-out form were visualized on the institutional website. All of data was obtained retrospectively and analyzed anonymously. All procedures were performed in accordance with the ethical standards of our institutional research committee and with the principles of the Declaration of Helsinki and were approved by the institutional ethics committees of Osaka City University Graduate School of Medicine (approval number: 3416) and Osaka City General Hospital (approved number: 2012139).

### Statistical analysis

Statistical analysis was performed by using JMP software (version 12.2.0; SAS Institute Japan, Tokyo, Japan). The Mann–Whitney U test was used to compare demographic characteristics (age and time since surgery), laboratory values, and hemodynamic data between patients with and without GEVs. Fisher’s exact test was performed to compare a demographic characteristic (male sex) and the CT findings between patients with and without GEVs. To clarify the factors predicting association of GEV, multivariate analyses were performed by using a logistic regression model on parameters that were selected in a univariate analysis (*P* < 0.1) between patients with and without GEVs. Odds ratios and a receiver operating characteristic (ROC) curve were generated, and the area under the ROC curve was calculated to identify the parameters and cut-off values for predicting association with GEV. To assess the accuracy of the cutoff of the platelet count generated from the imbalanced data comprising a small number of patients, the Matthews correlation coefficient (MCC) was calculated according to the following equation:

MCC=[(truepositive×truenegative)–(falsepositive×falsenegative)]/√[(truepositive+falsepositive)×(truepositive+falsenegative)×(truenegative+falsepositive)×(truenegative+falsenegative)].

All *P* values were two-sided and were considered significant when less than 0.05.

## Results

### Patient characteristics

The study population comprised 27 patients (median age, 14.8 years; range, 9.6 to 30.0 years) who had undergone the Fontan procedure. All patients were asymptomatic throughout follow-up and had never experienced episodes of gastrointestinal bleeding, ascites, edema, jaundice, encephalopathy, or hepatojugular reflex. The time since surgery, defined as the time between completion of the Fontan procedure and dynamic CT, was 13.7 ± 3.4 years (mean ± 1 SD; 95% confidence interval, 12.4 to 15.0 years). Patients’ baseline characteristics are shown in Table 1.

**Table 1:** Baseline characteristics and laboratory and CT findings in 27 patients who underwent Fontan surgery.

Age, mean ± 1 SD (range), years	17.2 ± 5.7 (9.6–30.0)
Male (%)	16 (59.2)
Diagnosis, n (%)	
TA	5 (18.5)
DORV	5 (18.5)
DORV, VSD	3 (11.1)
SV	2 (7.4)
DORV, SV	2 (7.4)
HRHS	2 (7.4)
PA, SV	2 (7.4)
DORV, HLHS	1 (3.7)
HLHS	1 (3.7)
PA, VSD	1 (3.7)
PA, ccTGA, VSD	1 (3.7)
PA, HLHS	1 (3.7)
VSD (type IV)	1 (3.7)
Type of Fontan procedure, n (%)	
TCPC-ECC	27 (100)
Fenestration present, n (%)	4 (14.8)
Time since surgery (for dynamic CT examination), mean ± 1 SD (range), years	13.7 ± 3.4 (7.2–20.6)
CVP, mean ± 1 SD (range), mm Hg	10.6 ± 3.4 (5–19)
Abdominal dynamic CT findings, n (%)	
Gastroesophageal varix	4 (14.8)
Gastric varix	3 (11.1)
Esophageal varix	1 (3.7)
Collateral veins	10 (37.0)
Gastrorenal shunt	5 (18.5)
Splenorenal shunt	4 (14.8)
Other collaterals	3 (11.1)
Spleen findings	
Splenomegaly	22 (81.5)
Asplenism	2 (7.4)
Polysplenism	0 (0)
Liver findings	
Hepatomegaly	27 (100)
Liver cirrhosis	3 (11.1)
Suspected focal nodular hyperplasia	14 (51.9)
Hepatocellular carcinoma	0 (0)
Porto-venous shunt	5 (18.5)
Arterio-portal shunt	0 (0)
Ascites	6 (22.2)

ccTGA, congenitally corrected transposition of the great arteries; CT, computed tomography; CVP, central venous pressure; DORV, double outlet right ventricle; ECC, extra-cardiac conduit; HLHS, hypoplastic left heart syndrome; HRHS, hypoplastic right heart syndrome; PA, pulmonary atresia; SV, single ventricle; TA, tricuspid atresia; TCPC, total cavopulmonary connection; VSD, ventricular septal defect.

### Development of GEVs and collateral veins after Fontan procedure

**Fig 2:**
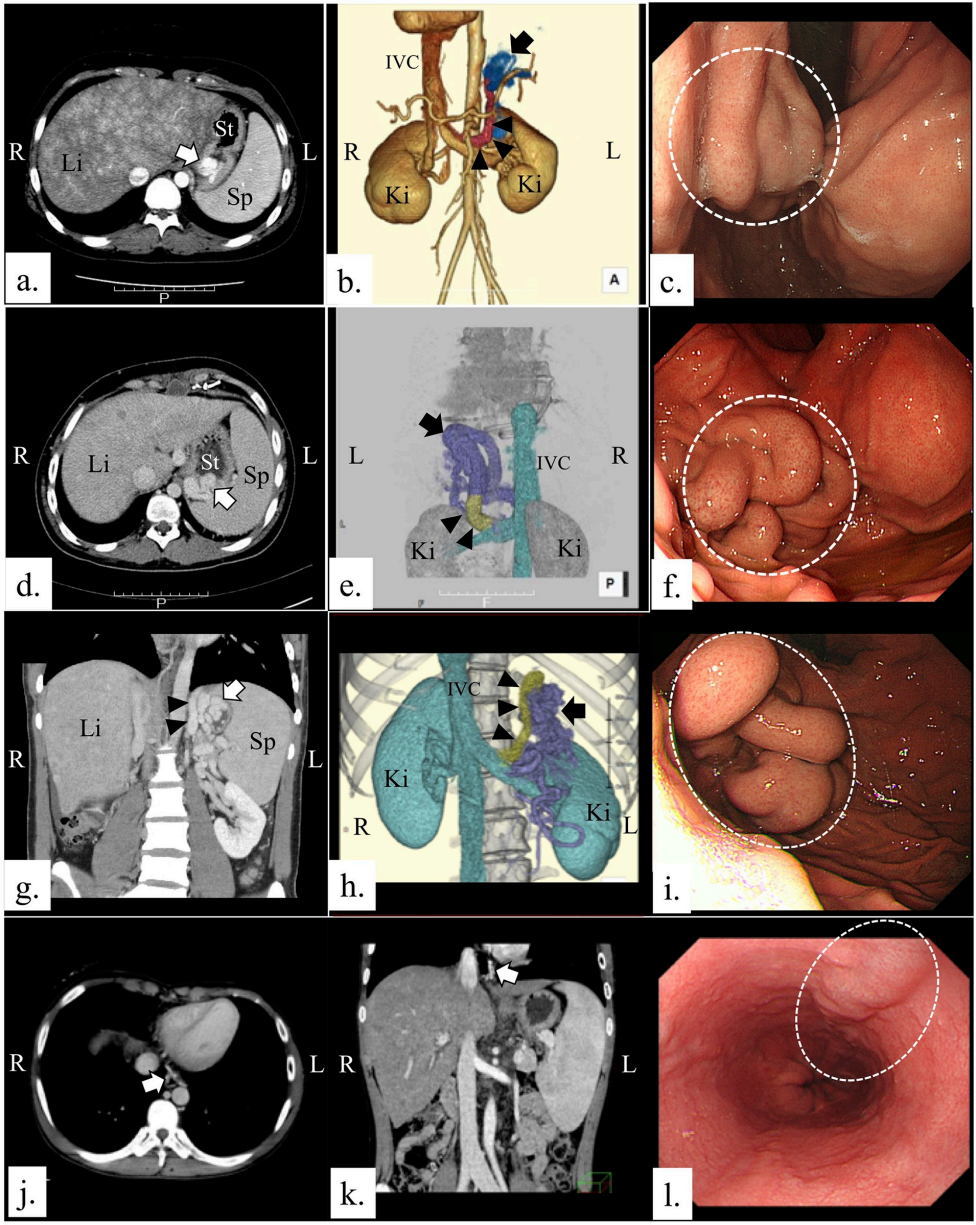
CT and endoscopic features of gastroesophageal varices in patients who underwent a Fontan procedure. (a, b) Abdominal dynamic CT images of a 19-year-old woman at 17 years after her Fontan procedure. (a) A portal phase image reveals a gastric varix (white arrow). (b) Three-dimensional CT image (anterior view) shows a gastric varix (blue, indicated by black arrow) draining into a gastrorenal shunt (red, indicated by black arrowheads). (c) Endoscopic image from the patient shown in Fig 2a and 2b. Esophagogastroduodenoscopy (EGD) reveals an enlarged nodular gastric varix (Lg-cf, F2, CW, RC0; indicated by dotted white circle). (d, e) Abdominal dynamic CT images of an 18-year-old man 14 years after his Fontan procedure. (d) Portal phase image reveals a gastric varix (indicated by white arrow). (e) Three-dimensional CT image (posterior view) shows a tortuous gastric varix (purple, indicated by black arrow) that drains into a gastrorenal shunt (yellow, indicated by black arrowheads). (f) Endoscopic image from the patient shown in Fig 2d and 2e. EGD reveals an enlarged nodular gastric varix (Lg-cf, F2, CW, RC0; indicated by dotted white circle). (g, h) Abdominal dynamic CT images of a 19-year-old man 13 years after a Fontan procedure. (g) Portal phase image reveals a gastric varix (indicated by white arrow) that drains into a gastrorenal shunt (indicated by black arrowheads). (h) Three-dimensional CT image (anterior view) shows a gastric varix (blue, indicated by black arrow) that drains into a gastrorenal shunt (red, indicated by black arrowheads). (i) Endoscopic image from the patient shown in Fig 2g and 2h. EGD reveals an enlarged nodular gastric fundal varix (Lg-f, F2, Cb, RC0) (indicated by dotted white oval). (j, k) Abdominal dynamic CT images of a 14-year-old boy 12 years after his Fontan procedure. The sagittal view (j) and coronal view (k) at the portal phase reveal a small esophageal varix (indicated by white arrows). (l) Endoscopic image of the patient shown in Fig 2j and 2k. EGD reveals a straight, small-caliber esophageal varix in the lower esophagus (Li, F1, Cw, RC0) (indicated by dotted white oval). GEV, gastroesophageal varix; IVC, inferior vena cava; Ki, kidney; L, left; Li, liver; R, right; Sp, spleen; St, stomach.

Abdominal dynamic CT disclosed GEVs in 14.8% (n = 4) of patients who had undergone the Fontan procedure; the GEVs consisted of three large gastric varices (11.1%) and one small esophageal varix (3.7%; Table 1 and Fig 2a, 2b, 2d, 2e, 2g, 2h, 2j and 2k). Esophagoduodenoscopy (EGD; Fig 2c, 2f, 2i and 2l) confirmed the GEVs found by dynamic CT, and all three of the enlarged and nodular gastric varices had indications for prophylactic therapy for primary variceal bleeding. In 10 patients (37.0%), dynamic CT revealed collateral veins, including gastrorenal shunts (18.5%, n = 5) and splenorenal shunts (14.8%, n = 4; Table 1). Of the four cases of gastrorenal shunt, three were associated with gastric varices as the draining veins (Fig 2b, 2e and 2h). In addition, CT disclosed splenomegaly in 81.5% (n = 22) and congenital asplenia in 7.4% (n = 2; Table 1) of the study population. Both of the patients with asplenia were free of GEVs. CT revealed hepatomegaly in all 27 patients, focal nodular hyperplasia in 51.9% (n = 14), and potential liver cirrhosis in 11.1% (n = 3; Table 1); no patients had indications of hepatocellular carcinoma (Table 1). Ascites was present in 22.2% (n = 6; Table 1) of the study population.

### Relationships between clinical, laboratory, hemodynamic, and CT findings and the presence of GEVs

The 27 patients were allocated to two groups according to the presence (n = 4) or absence (n = 23) of GEVs. None of the demographic characteristics (age, sex, and time since surgery) or central venous pressure differed between patients with and without GEVs (Table 2). Platelet counts were significantly lower, and hyaluronic acid and FIB-4 values were significantly higher, in patients with GEVs than in those without GEVs (see Table 2). Albumin concentration, ammonia concentration, MELD-XI score, and prothrombin time were similar between patients with and without GEVs (Table 2). In the CT findings, patients with GEVs were significantly more likely than those without GEVs to have gastrorenal shunts (Table 2).

**Table 2:** Comparison of demographic, laboratory, hemodynamic, and CT findings between Fontan patients with and without GEVs.

	Patients with GEV (n = 4)	Patients without GEV (n = 23)	*P*
Age, years (range)	17.9 ± 2.8 (13.8–19.8)	17.1 ± 6.1 (9.6–30.0)	0.71
Male (%)	3 (75)	13 (56.5)	0.62
Time since surgery, years (range)	14.2 ± 1.9 (12.2–16.8)	13.6 ± 3.6 (7.2–20.6)	0.64
Laboratory data			
Platelets, × 10^9^ /L	91 ± 25	180 ± 58	<0.001
AST, IU/L	26.5 ± 11.0	29.1 ± 8.7	0.68
ALT, IU/L	23.8 ± 13.4	22.5 ± 8.3	0.86
Total bilirubin, mg/dL	2.2 ± 1.6	1.0 ± 0.4	0.24
Albumin, g/dL	4.6 ± 0.1	4.5 ± 0.4	0.59
Prothrombin time, %	56.5 ± 26.2	70.0 ± 18.0	0.18
Ammonia, μmol/L	73.5 ± 48.4	42.7 ± 17.9	0.18
Hyaluronic acid, ng/mL	66.8 ± 5.3	45.3 ± 25.3	0.02
Type IV collagen 7s, ng/mL	9.8 ± 2.3	7.8 ± 1.6	0.26
MELD-XI	6.7 ± 4.1	2.5 ± 3.6	0.13
APRI	0.90 ± 0.27	0.54 ± 0.28	0.06
FIB-4	1.09 ± 0.23	0.66 ± 0.35	0.02
Hemodynamic data			
CVP, mmHg	12.3 ± 4.8	10.3 ± 3.2	0.47
Abdominal CT findings, n (%)			
Gastrorenal shunt	3 (75)	2 (8.7)	0.01
Splenorenal shunt	2 (50)	2 (8.7)	0.09
Splenomegaly	4 (100)	18 (78.2)	0.56
Asplenism	0 (0)	2 (8.7)	1.00
Hepatomegaly	4 (100)	23 (100)	1.00
Liver cirrhosis	1 (25)	2 (8.7)	0.39

ALT, alanine aminotransferase; APRI, AST-to-platelet ratio index; AST, aspartate aminotransferase; CT, computed tomography; CVP, central venous pressure; FIB-4, fibrosis-4 index; GEV, gastroesophageal varix.

Analysis was performed by using Mann–Whitney U test for demographic data (age and time since surgery), laboratory data, and hemodynamic data and by using Fisher’s exact test for demographic data (male sex) and CT findings.

Data are expressed as means ± 1 SD.

### Thrombocytopenia as a predictor of the presence of gastroesophageal varices

We performed univariate and multivariate analyses to clarify the parameters that were predictive of the presence of GEVs as a consequence of advanced portal hypertension. The results from the univariate regression analysis are shown in Table 3. Three variables—platelet count, APRI, and FIB-4 value—were used for multivariate analysis. Multivariate regression showed that platelet count was the only independent predictor of the presence of GEVs (Table 3). According to the area under the ROC curve, a platelet count of 119 × 10^9^/L highly specifically and sensitively predicted the presence of GEVs as a consequence of advanced portal hypertension (Table 4). Likewise, the maximum value in the MCC analysis (0.51) occurred at the platelet count cutoff point (119 × 10^9^ /L).

**Table 3:** Correlations between demographic and laboratory data and the presence of GEVs.

	*P* (univariate)	*P* (multivariate)
Age	0.81	
Time since surgery ^a^	0.74	
Platelet count	0.04	0.02
AST	0.59	
ALT	0.79	
Total bilirubin	0.15	
Hyaluronic acid	0.19	
Type IV collagen	0.11	
APRI	0.07	0.81
FIB-4	0.05	0.72

ALT, alanine aminotransferase; APRI, AST-to-platelet ratio index; AST, aspartate aminotransferase; FIB-4, fibrosis-4 index.

^a^ The time between surgery and dynamic CT.

**Table 4:** Platelet count to predict the presence of gastroesophageal varices.

Cutoff point	< 119 × 10^9^/L
AUROC	0.946
Sensitivity	100%
Specificity	87%
Accuracy	88.9%
Odds ratio (95% CI)	1.74 (1.18–3.85)
MCC	0.51

95% CI, 95% confidence interval; AUROC, area under the receiver operating characteristic curve; MCC, Matthews correlation coefficient.

## Discussion

Depending on the time since surgery, patients who have undergone a Fontan procedure are at risk of developing gastrointestinal and liver complications, including advanced liver fibrosis, cirrhosis, hepatocellular carcinoma, portal hypertension, protein-losing gastroenteropathy, and GEVs [5–15, 17, 18, 30, 31]. As these complications significantly influence patients’ quality of life after the surgery, numerous efforts have been made to develop and establish non-invasive and accurate methods or parameters to screen for them [7, 17, 32–35]. In particular, GEV is a life-threatening complication associated with cirrhosis and portal hypertension [19]. Patients who had undergone the Fontan procedure can develop cirrhosis subsequent to portal hypertension on a background of liver congestion [7, 30, 36]; they are therefore at risk of developing GEVs [7, 20, 21]. For example, in one of 44 patients who died after a Fontan procedure, the cause of death was esophageal variceal bleeding associated with cirrhosis [20]. In addition, GEVs were present in four of seven patients with cirrhosis, but four patients without cirrhosis lacked GEVs [7]. These findings therefore suggest that GEVs in patients who have undergone a Fontan procedure develop in association with cirrhosis. However, the overall prevalence of GEVs in this population is unclear, despite the known association of GEVs with cirrhosis. In the current study, we examined the usefulness of abdominal dynamic CT for GEV screening in patients who had undergone Fontan surgery, and we sought candidate non-invasive predictors of this complication. We found that 1) GEVs—notably gastric varices associated with gastrorenal shunts—are important long-term complications of the Fontan procedure and that 2) the platelet count can be used as a marker to predict the likelihood of GEV development in patients who have undergone a Fontan procedure.

We identified GEVs—especially gastric varices associated with gastrorenal shunts—in 11.1% of our patients. All of the gastric varices detected were moderately enlarged on EGD; this is an indication for prophylactic therapy for primary variceal bleeding. GEVs are life-threatening complications that cannot be ignored; therefore, patients who have undergone Fontan surgery should be screened for the presence of GEVs to optimize the timing of prophylactic therapy. In the current study, patients who had undergone a Fontan procedure typically had isolated gastric fundal varices rather than esophageal varices. Occurring in approximately 20% of patients with portal hypertension [37], gastric varices typically are less common than esophageal varices. For esophageal varices and gastric cardiac varices, the left gastric vein is the major afferent, whereas for fundal varices the short gastric vein and posterior gastric vein are the major afferents [38]. These pathophysiologic features suggest that Fontan-associated portal hypertension preferentially affects the short gastric vein and posterior gastric vein.

EGD is the “gold standard” screening method for GEVs [19]. As an alternative screening modality, abdominal dynamic CT is useful in detecting enlarged GEVs [7, 22, 23]. In addition, abdominal dynamic CT can reveal extraluminal pathology (e.g., gastrorenal shunts and cirrhosis); it can thus help to determine the GEV etiology (e.g., cirrhosis and portal hypertension) and reveal the vessels that supply and drain the GEV. In addition, using dynamic CT to assess gastrorenal shunts can guide decisions regarding the therapeutic approach (e.g., balloon-occluded retrograde transvenous obliteration or endoscopic sclerotherapy) to varices, because a gastrorenal shunt can be used to access gastric varices for performing balloon-occluded retrograde transvenous obliteration [39]. In our current study, all three enlarged gastric varices were associated with gastrorenal shunts; abdominal dynamic CT therefore played a critical role in the diagnosis of luminal and extraluminal pathology and in the selection of balloon-occluded retrograde transvenous obliteration via a gastrorenal shunt as the therapeutic strategy. Although EGD is necessary for the diagnosis and follow-up of GEVs, abdominal dynamic CT is a useful non-invasive screening tool for identifying GEVs and extraluminal pathology in patients who have undergone a Fontan procedure. In a meta-analysis, the sensitivity of CT for identifying esophageal and gastric varices was 89.6% and 95.5%, respectively [22]. In addition, CT is helpful in detecting ectopic varices.

Owing to the increased venous pressure after a Fontan procedure, the development of venovenous collateral vessels typically is common in these patients. However, in our study, patients with GEVs were significantly more likely than those without GEVs to have gastrorenal shunts. Gastrorenal shunts frequently become the draining routes of gastric varices [40], as occurred in the present cases. In contrast, splenorenal collateral veins generally do not communicate with the veins in the stomach and therefore do not form gastric varices [40]. Therefore, although collateral veins are more common after a Fontan procedure, the presence of gastrorenal shunts associated with gastric varices should also be considered as a notable feature in these patients.

Another important aspect regarding screening for GEVs—especially via abdominal dynamic CT—is when to perform the screening examination. Our findings suggest that the platelet count is the most valuable predictor of the need to perform GEV screening in patients who have undergone a Fontan procedure. In other words, an abnormally low platelet count (thrombocytopenia) may be helpful as a simple and convenient predictive parameter for decision-making regarding patients with GEVs. Typically, thrombocytopenia results from splenic sequestration as a complication of portal-hypertension-induced splenomegaly in chronic liver diseases [41, 42]. Therefore, the presence of thrombocytopenia in association with GEVs in patients who have undergone a Fontan procedure may explain the influence of portal hypertension as the etiology of GEVs. Numerous other studies have shown an association between thrombocytopenia (68 to 160 × 10^9^ /L) and the presence of GEV and have confirmed the high sensitivity of platelet counts for predicting GEV [43–45]. Despite our relatively small cohort, we likewise demonstrated a significant correlation between platelet count and the occurrence of GEVs and suggest that a platelet count lower than 119 × 10^9^ /L can be used as a marker of suspected GEV. Furthermore, the MCC increased to its maximum value of 0.51 at a platelet count of 119 × 10^9^/L; therefore, we consider that this cutoff value is appropriate and signals the need to evaluate patients for the presence of GEVs.

We acknowledge several limitations to our study. First, the study was a retrospective study comprising the small population, so the statistical results we obtained might have overestimated the patients’ risk of developing GEVs. However, despite the small population, we elucidated the clinical significance of GEV screening in patients who had undergone Fontan patients. Accurate details regarding the prevalence of GEVs and the relationship between various blood-biochemical and demographic parameters and the risk of GEV will be clarified in a large-cohort study based on the current results. Limitations to using CT in this patient population include adverse events such as radiation exposure, hypersensitivity reactions to contrast media, and the nephron injury and renal failure that contrast media can induce.

In conclusion, we found that GEVs are an important complication that can develop 10 years or more after Fontan surgery. To reveal GEVs and their draining collateral veins, abdominal dynamic CT may be a useful non-invasive modality for screening these patients, particularly those with thrombocytopenia (<119 × 10^9^/L).
